# Neonatal outcomes in Syrian and other refugees treated in a tertiary hospital in Turkey

**DOI:** 10.3906/sag-1806-86

**Published:** 2019-06-18

**Authors:** İstemi Han ÇELİK, Zehra ARSLAN, Dilek ULUBAŞ IŞIK, Ömer Lütfi TAPISIZ, Leyla MOLLAMAHMUTOĞLU, Ahmet Yağmur BAŞ, Nihal DEMİREL

**Affiliations:** 1 Division of Neonatology, Department of Pediatrics, University of Health Sciences,Etlik Zübeyde Hanım Women’s Health Teaching and Research Hospital, Ankara Turkey; 2 Department of Obstetrics and Gynecology, University of Health Sciences,Etlik Zübeyde Hanım Women’s Health Teaching and Research Hospital, Ankara Turkey; 3 Division of Neonatology, Department of Pediatrics, School of Medicine, Yıldırım Beyazıt University, Ankara Turkey

**Keywords:** Neonatal outcomes, refugees, Syrian

## Abstract

**Background/aim:**

Turkey accepts refugees from many countries, principally Syria. More than 2.7 million refugees live in Turkey.****We evaluated the neonatal outcomes of refugees.

**Materials and methods:**

We retrospectively reviewed the clinical and demographic characteristics of refugee infants born in our hospital between August 2013 and September 2016.

**Results:**

Refugees (718 Syrian, 136 Iraqi, 32 Afghani, and 21 of other nationalities) accounted for 907 of 49,413 births. The mean refugee maternal age was lower than that of Turkish women, whereas the gestational age (GA) and birthweight were similar. Refugees required fewer cesarean sections but exhibited greater small- and large-for-GA rates (P < 0.05). Refugee and Turkish infant mortality rates did not differ significantly (0.8 vs. 0.4%). Eighty-nine (12.3%) refugee neonates and 6682 (13.5%) Turkish neonates were admitted to our neonatal intensive care unit (NICU). Jaundice and perinatal asphyxia were significantly more common in refugees, whereas respiratory distress syndrome, GA ≤32 weeks, and infant birthweight <2000 g were more common in Turkish infants. The total NICU admission cost of approximately 450,000 USD was paid by the Turkish government.

**Conclusion:**

The numbers of refugees and refugee births continue to grow. The Turkish people and government have provided medical, social, and economic support to date; international assistance is needed.

## 1. Introduction

The United Nations High Commission for Refugees reported that global populations of concern and refugees numbered approximately 55 and 14 million, respectively, in January 2016 [1]. The Syrian conflict, which commenced in March 2011, is still ongoing. Since 2011, 4.8 million Syrians have become refugees in neighboring countries, principally Turkey, Lebanon, and Jordan [2]. Turkey is the major host country; more than 2.7 million refugees live in Turkey [2,3]. Most (about 2.4 million) are not in camps but are spread throughout Turkey from southern to central and western cities including İstanbul, Ankara, and İzmir (the largest Turkish cities); 255,000 refugees live in camps at sites on the Syrian border [4]. Turkey accepts refugees from many countries, including Iraq, Afghanistan, Pakistan, and Central Asian countries. Turkey hosts over 300,000 non-Syrian refugees [5]. Turkey has the largest refugee population worldwide. Only 60% of refugees can benefit from Turkish healthcare services because of lack of information, communication barriers, or lack of identification cards issued by the Turkish government [6].

Women in conflict areas experience poor prenatal and natal outcomes (maternal and perinatal mortality, low birthweight (BW), preterm delivery, and infant congenital anomalies). In this study, we compared the neonatal outcomes of refugee and Turkish newborns.

## 2. Materials and methods

This retrospective study was performed at the University of Health Sciences Etlik Zübeyde Hanım Women’s Health Teaching and Research Hospital between August 2013 and September 2016. Our hospital, located in Ankara, is one of the largest women’s health training and research hospitals in Turkey. This study was approved by our local ethics committee on 10 June 2016 (Approval No. 90057706-900/EGITIM-143).

The demographic characteristics of refugees and Turkish newborns were retrieved from medical records; we recorded nationality, sex, gestational age (GA), maternal age, maternal or gestational disease status, mode of delivery, BW, BW small-for-GA (SGA), BW large-for-GA (LGA), and the need for admission to our neonatal intensive care unit (NICU). Neonatal morbidities were defined as respiratory distress syndrome (RDS), transient tachypnea of the newborn (TTN), sepsis, jaundice, polycythemia, hypoglycemia, perinatal asphyxia, feeding difficulties, and prolonged hospitalization. Congenital anomalies and neonatal mortality rates were recorded.

Respiratory distress requiring NICU admission was any respiratory morbidity irrespective of diagnostic test results or the need for therapeutic intervention. We admitted patients with RDS, TTN, meconium aspiration syndrome (MAS), and pneumonia. We also admitted infants with clinically apparent jaundice after laboratory measurement of the serum bilirubin concentration by reference to the American Academy of Pediatrics guidelines [7]. We admitted infants with sepsis who exhibited at least three of the following symptoms: bradycardia or tachycardia, hypotonia, hypotension, cyanosis, apnea, respiratory distress, tachypnea, poor skin color and/or perfusion, feeding difficulty, irritability, lethargy, elevated acute-phase reactance, and a positive blood culture. We admitted those with perinatal asphyxia: Apgar score <5 at 5 and 10 min, umbilical artery pH <7.0, and/or a base deficit of ≥12 mmol/L. The admitted hypoglycemic infants had serum glucose concentrations of <45 mg/dL over the first 24 h and <50 mg/dL thereafter. Infants admitted with feeding difficulties were unable to breast- or bottle-feed, exhibited abdominal distension, or were vomiting. Admitted SGA infants had BW below the 10th percentile for their GA, and LGA infants exhibited BW above the 90th percentile on Fenton’s growth curve [8]. Admitted infants with polycythemia had venous hematocrit of >70% or else >65% accompanied by hypoglycemia, feeding difficulty, and/or hypotonia. We admitted low-BW (LBW) and extremely low-BW (ELBW) infants (<2500 g and <1000 g, respectively), those born after preterm premature membrane rupture prior to 37 weeks’ GA, those with bronchopulmonary dysplasia (BPD) (oxygen dependence at a postmenstrual age of 36 weeks in those of GA <32 weeks), and those with necrotizing enterocolitis diagnosed using the modified Bell criteria. 

### 2.1. Statistical analysis

All data were analyzed using SPSS 21.0 for Mac (IBM Corp., Armonk, NY, USA). Student’s t-test and the Mann–Whitney U test were employed to compare continuous parametric and nonparametric variables. The Kruskal–Wallis test was used to compare more than two independent groups. The chi-square test was employed to compare categorical variables. Data are expressed as mean ± standard deviation, median (minimum–maximum), or percentage; P < 0.05 was considered to reflect statistical significance.

## 3. Results

There were 49,413 births during the study period, of which 907 (1.83%) were refugee births; the latter group increased annually from 2013 to 2016: 17.2, 89, 250, and 310 per 10,000 births. In terms of nationality, the refugees from Syria, Iraq, Afghanistan, and other countries (Iran, Sudan, Somalia, Ethiopia, and Turkmenistan) giving birth numbered 718, 136, 32, and 21, respectively. Totals of 26.4%, 36%, and 43.8% of Syrian, Iraqi, and Afghani women made at least one antenatal visit, over half of which were to our hospital, while 71.6% of refugees had no antenatal follow-up. Table 1 shows the demographic characteristics of the patients. The median refugee maternal age was significantly lower than that of Turkish women [23 (14–45) vs. 27 (15–45) years, respectively]. Adolescent pregnancy was significantly higher in the refugee group (17% vs. 3%), whereas higher maternal age was significantly higher in the Turkish population (13.1% vs. 7.2%). The median GA and BW of refugee and Turkish infants were similar. The median gravidities of Syrian and Iraqi mothers were 2 (1–12) and 3 (1–7); the parities were 2 (1–11) and 2 (1–7), respectively. Grand multiparity rate of refugees was 14%. Cesarean section (CS) was required by 36.3% of refugees, a significantly lower proportion than that for Turkish women (43.9%). Indications for CS in refugees included previous CS (44.7%), fetal distress (20.8%), and preeclampsia, cephalopelvic disproportion, or placental anomalies (34.5%). The rates of preterm birth in refugees and Turkish women were similar (19.3 vs. 17.9%). Antenatal antibiotic treatment was employed in 36 refugees with PPROM and clinical chorioamnionitis. The antenatal betamethasone application rates of refugees and Turkish women were similar. The SGA and LGA rates of refugees and Turkish women were significantly different at 15.7% vs. 9.7% and 2.5% vs. 1.4%, respectively. Low birth weight rates of Syrian and all refugees were 13.9% and 12.5%, respectively. The mean body mass index of refugee mothers was 27 ± 4.1 kg/m2 and did not differ by nationality. The mortality rate of refugee infants was similar to that of Turkish infants (0.8% vs. 0.4%).

**Table 1:** Demographic characteristics of refugee and Turkish neonates.

	Syrian(718)	Iraqi(136)	Refugees(907)	Turkish(48,506)	
Maternal age	23 (14–45)	26 (16–42)	23 (14–45)	27 (15–45)	P < 0.051
Gestational age, weeks	38 (25–42)	38 (29–42)	38 (25–42)	38 (23–41)	P > 0.05
Birth weight, g	2998 ± 547	3099 ± 540	3027 ± 548	3010 ± 741	P > 0.05
Sex, male, n (%)	389 (52.6%)	72 (52.9%)	476 (52.5%)	26726 (55.1%)	P > 0.05
Vaginal delivery, n (%)	456 (61.8%)	92 (67.6%)	577 (63.7%)	27217 (56.1%)	P < 0.052
SGA, n (%)	119 (16.1%)	22 (16.3%)	142 (15.7%)	4737 (9.7%)	P < 0.053
LGA, n (%)	16 (2.2%)	4 (3%)	23 (2.5%)	722 (1.4%)	P < 0.054
Consanguineous marriages, n (%)	126 (17.5%)	36 (26.4%)	168 (18.5%)	-	P < 0.055
Preterm birth, <37 GA, n (%)	139 (19.3%)	24 (17.6%)	172 (18.9%)	8682 (17.9%)	P > 0.05
Antenatal steroids, <34 GA, n (%)	39/54 (72%)	8/9 (88%)	48/64 (75%)	2512 (70%)	P > 0.05
Congenital malformation, n (%)	5 (0.6%)	2 (1.4%)	7 (0.8%)	-	-
Mortality, n (%)	6 (0.8%)	-	6 (0.6%)	210 (0.4%)	P > 0.05

1 Turkish vs. Syrian, Iraqi, and all refugees. 2 Turkish vs. Syrian, Iraqi; Turkish vs. all refugees. 3,4 Turkish vs. all refugees. 5 Iraqi vs. Syrian. Only Syrian and Iraqi neonates were compared with Turkish neonates because of small case numbers of other nationalities. SGA, Small for gestational age; LGA, large for gestational age; GA, gestational age.

Table 2 shows the NICU admission diagnoses of refugee and Turkish neonates. Totals of 110 (12.3%) refugee and 6682 (13.5%) Turkish neonates were admitted to the NICU; the refugee numbers increased as the birth numbers increased, with annual rates of 0/11 (0%), 14/145 (9.6%), 32/413 (7.7%), and 64/338 (18.9%) from 2013 to 2016, respectively. Most admissions were for treatment of RDS, TTN, jaundice, perinatal asphyxia, GA <32 weeks, BW <2000 g, feeding difficulties, and hypoglycemia. The rates of jaundice and perinatal asphyxia were significantly higher in refugees, whereas the rates of RDS, GA <32 weeks, and BW <2000 g were higher in Turkish infants. 

**Table 2:** Admission diagnosis of refugee and Turkish neonates.

	Syrian(718)	Iraqi(136)	Refugees(907)	Turkish(48,506)	
NICU admission, n (%)	89 (12.3%)	18 (13.2%)	110 (12.1%)	6682 (13.5%)	P > 0.05
RDS, n (%)	15 (16.9%)	1 (5.6%)	16 (14.5%)	1380 (20.6%)	P < 0.051
TTN, n (%)	23 (25%)	5 (27.7%)	28 (25.4%)	1388 (20.7%)	P > 0.05
Jaundice, n (%)	24 (26.9%)	3 (16.6%)	29 (26.9%)	1050 (15.7%)	P < 0.052
Perinatal asphyxia, n (%)	3 (3.3%)	-	3 (2.7%)	37 (0.5%)	P < 0.053
≤32 GA or low birth weight, n (%)	15 (16.8%)	3 (16.6%)	18 (16.3%)	1816 (27%)	P < 0.054
Feeding difficulty, n (%)	-	2 (11%)	2 (1.8%)	111 (1.6%)	P > 0.05
Hypoglycemia, n (%)	2 (2.2%)	1 (5.5%)	3 (2.7%)	122 (1.6%)	P > 0.05

1–4 Turkish vs. Syrian, refugees. NICU, Neonatal intensive care unit; RDS, respiratory distress syndrome; TTN, transient tachypnea of newborn; GA, gestational age.

Refugee morbidities during NICU admission are shown in Table 3. The rates of RDS, surfactant use, TTN, sepsis, hypoglycemia, hypocalcemia, jaundice, dehydration, rehospitalization, and breastfeeding were similar between Syrian and Iraqi neonates. The median duration of hospital stay was significantly higher for Iraqi than for Syrian neonates (7.5 vs. 4 days). When the morbidities of refugee and Turkish neonates of ≤32 GA were compared, the rates of RDS (79% vs. 76%), sepsis (20.8% vs. 16.3%), and necrotizing enterocolitis (0% vs. 1.4%) were similar but the rates of surfactant use (25% vs. 60%) and BPD (4.2% vs. 22.5%) were significantly higher in Turkish neonates. The total NICU admission cost of refugee neonates, which was covered by the Turkish government, was approximately 450,000 USD over the study period. 

**Table 3:** NICU morbidities of refugee neonates.

	Syrian(89)	Iraqi(18)	Refugees (110)	
RDS, n (%)	26 (29%)	7 (38.8%)	33 (30%)	P > 0.05
Surfactant use, n (%)	5/26 (19.2%)	2/7 (28.5)	7/33 (21%)	P > 0.05
TTN, n (%)	26 (29.2%)	5 (27.7%)	31 (28.1%)	P > 0.05
Sepsis, n (%)	5 (5.6%)	2 (11.1%)	7 (6.3%)	P > 0.05
Hypoglycemia, n (%)	11 (12.3%)	1 (5.6%)	12 (10.9%)	P > 0.05
Jaundice, n (%)	59 (66.3%)	14 (77.7%)	75 (68.1%)	P > 0.05
Necrotizing enterocolitis, n (%)	1 (1.1%)	-	1 (0.9%)	-
Feeding intolerance, n (%)	1 (1.1%)	3 (16.6%)	4 (3.6%)	P < 0.051
Polycythemia, n (%)	6 (6.7%)	-	6 (5.4%)	-
Dehydration, n (%)	3 (3.3%)	1 (5.6%)	4 (3.6%)	P > 0.05
Rehospitalization, n (%)	6 (6.7%)	1 (5.6%)	8 (7.2%)	P > 0.05
Breastfeeding, n (%)	95%	95%	95.8%	P > 0.05
Duration of hospital stay, days	4 (1–76)	7.5 (2–75)	4 (1–76)	P < 0.052

1 Iraqi vs. Syrian. 2 Iraqi vs. Syrian. NICU, Neonatal intensive care unit; RDS, respiratory distress syndrome; TTN, transient tachypnea of newborn.

## 4. Discussion

The number of refugee births is increasing significantly in Turkey. Since 2011, 177,568 Syrian neonates have been born, including 70,000 in 2015 [9]. Thus, the Syrian population will soon account for more than 5% of all deliveries in Turkey. During our study period, Syrian births accounted for 1.48% of all births; the figure in 2016 was 2.6%. In another tertiary care unit in Ankara, 1.2% of all births in 2013 and 2014 were to Syrian mothers [10]. Refugee healthcare costs are covered by the Turkish government, which has spent 30 billion USD to date to care for Syrian refugees [https://www.aa.com.tr/tr/ekonomi/basbakan-yardimcisi-akdag-suriyeliler-icin-harcanan-toplam-maliyet-84-milyar-880-milyon-lira/990509]. Healthcare coverage including protective healthcare, inpatient and outpatient clinic costs, and drug costs is provided to refugees after application to the immigration administration in Turkey.

Most clinical and demographic characteristics (including GA and BW) of refugee neonates were similar to those in Turkish neonates. This may be associated with better antenatal care during risky pregnancies and the better socioeconomic status of refugees admitted to our hospital. Most of these refugees live in and around Ankara rather than in camps in the south and east of Turkey. Most of our refugees can afford to live outside the camps or with relatives. Ankara has many tertiary obstetric and neonatology clinics, and refugees are seen free of charge. However, it should be noted that most of the refugees do not have adequate antenatal follow-up and we do not know the exact number of them because of communication problems. Translators have been employed in our hospital for at least 3 years to solve these communication problems. The refugee maternal age was younger than that of Turkish women, reflecting sociocultural features and the idea that marriage might protect against war and related problems. Adolescent pregnancy was higher in Syrian refugees in our study and this finding is consistent with Erenel et al.’s study (14.2% vs. 5.3%) [11].

The CS rate in refugee mothers was 36.3%, lower than that in Turkish mothers (43.9%). Although the rate of primary CS was lower in refugees, the rate of previous CS was similar. Huster et al. [12] reported a CS rate of 35% among 6366 deliveries of Syrian refugees in Lebanon. The rate in another study by Huster was higher than that in our study (57% vs. 44%), and the CS rate in Syria was reported as 12%–15% before the war [13]. Recent studies from Turkey reported CS rates of Syrian refugees and Turkish citizens similar to our study as 32.3% vs. 43.1% and 30% vs. 44% [11,14]. It should be noted that the CS rate in Turkey has decreased over time. The rate of refugee antenatal evaluation seems low, but often, medical providers could not take antenatal histories because most refugees cannot speak either Turkish or English. We suggest that the antenatal evaluation rate was actually higher than our data indicate. In a study from Lebanon, only 50% of pregnant women received two or more antenatal evaluations [15]. The rate of preterm birth among Syrian refugees was similar to that of Turkish women (19.3% vs. 17.9%); Buyuktiryaki et al. [10] reported a rate of 26%. In the cited work, the numbers of LBW and ELBW infants were 10 and 4; our figures were 96 and 5. The NICU admission rate in the cited work was lower than that in our study [10.9% (50/457) vs. 12.3% (89/718)]. In a study in Syria, Wannous et al. [16] found that the incidence of LBW was 6.6% and that of preterm birth was 20% in 10,585 neonates, but infants were included in the preterm birth group up to 37 GA. Low birth rate of Syrian refugees in our study was 13.9%, which was higher than in Wannous et al.’s study. This may be associated with the socioeconomic status of refugees in Turkey. Erenel et al. reported the LBW rate of Syrian refugees after excluding preterm births as 6.8% in İstanbul, but if preterm births were included the LBW rate was calculated as 17.3%, higher than in our study. Masterson et al. [17] reported LBW and preterm birth rates of 10.5% and 26.5% among 34 births to Syrian refugees in Lebanon. We gave antenatal steroids to refugee mothers with infants of <34 weeks of GA of age (75%), which may have been associated with better than expected neonatal outcomes. The birthweight of Syrian refugees was 2998 ± 547 g in our study while it was reported as 3099 ± 525 and 3110 (540–4790) g in two studies from İstanbul, Turkey [11,14]. The twin pregnancy rate was higher in the work of Wannous et al. [16] than in our study (9.2% vs. 2.2%).

The incidence of SGA in the United States was 10.2% of all births in 2005 [18]. We previously found that the SGA rates in premature infants of ≤32 weeks of GA and term infants were 8.7% and 9.7%, respectively [19,20]. In the present study, the SGA (15.7%) and LGA (2.5%) rates of refugees were significantly higher than those of the Turkish population (9.7%), possibly due to poor antenatal care. Bouchghoul et al. [21] found a lower SGA rate in the Zaatari Refugee Camp, Jordan (5.1%; 4/299). The CS, respiratory distress, jaundice, and sepsis rates were 5.4%, 1%, 0.3%, and 0.3%, respectively. The low morbidity and SGA rates were associated with the referral of 82 pregnant women to tertiary care units.

The NICU admission rates of refugee and Turkish infants were similar. The perinatal asphyxia rate was significantly higher among Syrian infants, perhaps associated with poor antenatal evaluation, late admission to the hospital, and communication problems during admission and birth. The jaundice rate was also higher in refugee infants. Inadequate feeding education due to communication problems and an inability to visit after discharge may explain this observation. The mortality rates of refugee and Turkish infants were similar (0.8% vs. 0.4%) and were lower than that of the study of Buyuktiryaki et al. [10] (1.8%). Most of the infants who died had congenital malformations and were extremely premature. The World Health Organization reported Turkish and Syrian neonatal mortality rates of 0.4% and 0.8% before the Syrian crisis began [22,23]. This may reflect better healthcare in Turkey and the acceptance of Syrian refugees into our healthcare system. Most morbidities during NICU stay did not differ among refugees from different countries, reflecting their similar lifestyles and healthcare coverage in Turkey. Among infants of GA of ≤32 weeks, more Turkish neonates than refugee neonates exhibited developmental immaturity, explaining the higher rates of surfactant use and BPD in immigrant infants.

Our study has certain limitations. First, the study was conducted in a tertiary hospital with world-class obstetrics and neonatology departments, where refugees may receive better care than is available in camps. As discussed above, the socioeconomic conditions of refugees admitted to our hospital may be better than those of refugees living elsewhere in Turkey, explaining why their outcomes were similar to those of the Turkish population. Our results may not reflect the neonatal outcomes of refugees living in other parts of the country.

In conclusion, Turkey continues to accept refugees from various countries, especially Syria, and annual refugee births are increasing. These infants have an impact on the healthcare system, the economy, and the sociocultural life of Turkey. Refugees require healthcare and social support, improving both maternal and neonatal care. The Turkish people and government have long provided medical, social, and economic support, but international assistance is needed as well. Refugees living in other Turkish cities may differ in terms of maternal and neonatal morbidities from our patients, reflecting their socioeconomic conditions; further studies are needed.

## References

[ref0] (2016). United Nations High Commissioner for Refugees. UNHCR Global Appeal.

[ref1] (2018). United Nations High Commissioner for Refugees. Syria Regional Refugee Response.

[ref2] (2016). Regional Refugee and Resilience Plan.

[ref3] (2017). Current Status in AFAD Temporary Protection Centres.

[ref4] (2018). Humanitarian Aid and Civil Protection.

[ref5] (2013). Republic of Turkey Prime Ministry Disaster and Emergency Management Presidency.

[ref6] (2004). American Academy of Pediatrics Subcommittee on Hyperbilirubinemia. Management of hyperbilirubinemia in the newborn infant 35 or more weeks of gestation. Pediatrics.

[ref7] (2013). A systematic review and meta-analysis to revise the Fenton growth chart for preterm infants. BMC Pediatrics.

[ref8] (2016).

[ref9] (2015). Neonatal outcomes of Syrian refugees delivered in a tertiary hospital in Ankara, Turkey. Conflict and Health.

[ref10] (2017). Clinical characteristics and pregnancy outcomes of Syrian refugees: a case-control study in a tertiary care hospital in Istanbul, Turkey. Archives of Gynecology and Obstetrics.

[ref11] (2014). Cesarean sections among Syrian refugees in Lebanon from December 2012/January 2013 to June 2013: probable causes and recommendations. Yale Journal of Biology and Medicine.

[ref12] (2009). Hospital-based caesarean section in the Arab region: an overview. Eastern Mediterranean Health Journal.

[ref13] (2017). Birth characteristics of Syrian refugees and Turkish citizens in Turkey in 2015. International Journal of Gynecology & Obstetrics.

[ref14] (2015). An assessment of antenatal care among Syrian refugees in Lebanon. Conflict and Health.

[ref15] (2001). and determinants of low birth weight in Syrian government hospitals. Eastern Mediterranean Health Journal.

[ref16] (2014). Assessment of reproductive health and violence against women among displaced Syrians in Lebanon. BMC Women’s Health.

[ref17] (2005). Centers for Disease Control.

[ref18] (2014). Risk of retinopathy of prematurity in small for gestational age premature infants. Indian Pediatrics.

[ref19] (2015). Early-term delivery and adverse neonatal outcomes at a tertiary center in Turkey. Turkish Journal of Pediatrics.

[ref20] (2015). Humanitarian obstetric care for refugees of the Syrian war. The first 6 months of experience of Gynecologie Sans Frontieres in Zaatari Refugee Camp (Jordan). Acta Obstetricia et Gynecologica Scandinavica.

[ref21] (2013). Ministry of Health of Turkey.

